# First report and multilocus genotyping of *Enterocytozoon bieneusi* from Tibetan pigs in southwestern China

**DOI:** 10.1051/parasite/2019021

**Published:** 2019-05-01

**Authors:** Run Luo, Leiqiong Xiang, Haifeng Liu, Zhijun Zhong, Li Liu, Lei Deng, Ling Liu, Xiangming Huang, Ziyao Zhou, Hualin Fu, Yan Luo, Guangneng Peng

**Affiliations:** 1 The Key Laboratory of Animal Disease and Human Health of Sichuan Province, College of Veterinary Medicine, Sichuan Agricultural University Chengdu Sichuan Province 611130 PR China; 2 Chengdu Giant Panda Breeding Research Base Chengdu Sichuan Province 625001 PR China

**Keywords:** *Enterocytozoon bieneusi*, Tibetan pigs, ITS gene, Multilocus genotype

## Abstract

*Enterocytozoon bieneusi* is a common intestinal pathogen in a variety of animals. While *E. bieneusi* genotypes have become better-known, there are few reports on its prevalence in the Tibetan pig. This study investigated the prevalence, genetic diversity, and zoonotic potential of *E. bieneusi* in the Tibetan pig in southwestern China. Tibetan pig feces (266 samples) were collected from three sites in the southwest of China. Feces were subjected to PCR amplification of the internal transcribed spacer (ITS) region. *Enterocytozoon bieneusi* was detected in 83 (31.2%) of Tibetan pigs from the three different sites, with 25.4% in Kangding, 56% in Yaan, and 26.7% in Qionglai. Prevalence varies according to age group, from 24.4% (age 0–1 years) to 44.4% (age 1–2 years). Four genotypes of *E. bieneusi* were identified: two known genotypes EbpC (*n* = 58), Henan-IV (*n* = 24) and two novel genotypes, SCT01 and SCT02 (one of each). We compare our results with a compilation of published results on the host range and geographical distribution of *E. bieneusi* genotypes in China. Phylogenetic analysis showed these four genotypes clustered to group 1 with zoonotic potential. Multilocus sequence typing (MLST) analysis of three microsatellites (MS1, MS3, MS7) and one minisatellite (MS4) was successful in 47, 48, 23 and 47 positive specimens and identified 10, 10, 5 and 5 genotypes at four loci, respectively. This study indicates the potential danger of *E. bieneusi* to Tibetan pigs in southwestern China, and offers basic advice for preventing and controlling infections.

## Abstract (fr)

*Enterocytozoon bieneusi* est un agent pathogène intestinal commun chez de nombreux animaux. Bien que les génotypes d’*E. bieneusi* soient mieux connus, il existe peu de rapports sur sa prévalence chez le porc tibétain. Cette étude a examiné la prévalence, la diversité génétique et le potentiel zoonotique d’*E. bieneusi* chez le porc tibétain du sud-ouest de la Chine. Des matières fécales de porcs tibétains (266 échantillons) ont été collectées sur trois sites dans le sud-ouest de la Chine. Les matières fécales ont été soumises à une amplification PCR de la région de l’espaceur interne transcrit (ITS). *Enterocytozoon bieneusi* a été détecté chez 83 (31,2 %) des porcs tibétains des trois sites différents, dont 25,4 % à Kangding, 56 % à Yaan et 26,7 % à Qionglai. La prévalence varie selon le groupe d’âge, de 24,4 % (0 à 1 an) à 44,4 % (1 à 2 ans). Quatre génotypes d’*E. bieneusi* ont été identifiés : deux génotypes connus, EbpC (*n* = 58), Henan-IV (*n* = 24) et deux nouveaux génotypes, SCT01 et SCT02 (un cas de chaque). Nous comparons nos résultats avec une compilation des résultats publiés sur les hôtes et la répartition géographique des génotypes d’*E. bieneusi* en Chine. L’analyse phylogénétique a montré que ces quatre génotypes étaient regroupés dans le groupe 1, avec potentiel zoonotique. L’analyse par typage de séquence multilocus (MLST) de trois microsatellites (MS1, MS3, MS7) et d’un minisatellite (MS4) a été réussie chez 47, 48, 23 et 47 spécimens positifs et a identifié 10, 10, 5 et 5 génotypes à quatre loci, respectivement. Cette étude indique le danger potentiel d’*E. bieneusi* pour les porcs tibétains du sud-ouest de la Chine et propose des conseils de base pour la prévention et le contrôle des infections.

## Introduction

Microsporidia are obligate intracellular eukaryotic pathogens, classified as fungi, which are composed of approximately 1300 species in 160 genera [7]. To date, 17 microsporidia species are known to infect humans, and of these, *Enterocytozoon bieneusi* is the most prevalent, accounting for over 90% of cases of human microsporidiosis [6]. Since its first detection in an HIV/AIDS patient in 1985, a growing body of literature attests to *E. bieneusi* expanding its range of hosts [47, 51, 52]. Infection of healthy individuals with *E. bieneusi* results in self-limiting diarrhea and malabsorption. However, this pathogen can cause life-threatening diarrhea in immunocompromized individuals, such as AIDS patients and transplant recipients [35]. Normally, fecal-oral routes serve as the main infection pathways in humans and animals, while human inhalation of *E. bieneusi* spores has also been documented [54, 58]. PCR-based molecular techniques may be used to analyze the *E. bieneusi* genome, and for diagnosis. Based on the nested PCR amplification of internal transcribed spacers (ITS) of small subunits of ribosomal rRNA (SSU rRNA), over 240 *E. bieneusi* genotypes have been identified globally [5, 56, 59]. Phylogenetic analysis reveals that these genotypes clustered into nine groups. Group 1 is considered zoonotic, and is composed of genotypes from humans and a few animals, while groups 2–9 have particular host associations or are found in wastewater [5, 51]. To better comprehend *E. bieneusi* genetic diversity and molecular characteristics, high-resolution multi-locus sequence typing (MLST) using three microsatellites (MS1, MS3 and MS7) and one minisatellite (MS4) as markers was used to explore genotype taxonomy and transmission routes [9, 55, 56].

In the southwest of China, Tibetan pigs are widely kept for livelihood and are economically important for farmers, especially on the plateau. Tibetan pigs have firm black hair which differs from that of the common pig, and they are sturdy, outdoor foragers. They may act as reservoirs for *E. bieneusi* spores and zoonotic transmission of disease. Although much research has been carried out on *E. bieneusi* [10, 28, 30], few studies have examined its epidemiology or Tibetan pig-associated genomes in China [20, 57]. Tibetan pigs in southwestern China have been entirely unstudied. Therefore, this study aimed to establish the incidence and molecular characteristics of *E. bieneusi* in Tibetan pigs, to use ITS and MLST to evaluate its genetic diversity, and to assess the potential for zoonotic transmission of microsporidiosis between Tibetan pigs and humans.

## Materials and methods

### Ethics statement

The study was conducted in accordance with the Research Ethics Committee and the Animal Ethics Committee of Sichuan Agricultural University. Prior to fecal specimen collection, permission was obtained from the keepers of the animals whenever possible.

### Collection of Tibetan pig fecal specimens

Fresh fecal specimens were collected from 266 Tibetan pigs during June–October 2017. Samples were obtained mainly from three cities in Sichuan province, southwestern China, including Yaan (*n* = 50) (29°98′S, 103 °E), Kangding (*n* = 201) (30°05′S, 101°4′E), and Qionglai (*n* = 15) (30°42′S, 103°47′E) (Table 1). Kangding is located in a subtemperate plateau humid climate zone; Yaan and Qionglai have a subtropical humid monsoon climate and these special environments are beneficial to rear Tibetan pigs. Three farms applied intensive feeding conditions and had excellent hygiene conditions. The breeding density of Tibetan pigs in Kangding was higher than in other cities. From each farm, samples were randomly collected from at least 15% of the animals. The ages of Tibetan pigs sampled ranged from 1 to 2 years. Each specimen (approximately 200 mg) was collected using sterile disposable latex gloves immediately after being defecated onto the ground, and transferred into 50 mL plastic containers. Meanwhile, the age, gender, geographic origin, number and date of each sample was also recorded. No experimental Tibetan pigs showed diarrheic or gastrointestinal conditions. Samples were stored at 4 °C in 2.5% (w/v) potassium dichromate.

**Table 1.** Factors associated with prevalence of *Enterocytozoon bieneusi* in Tibetan pigs in southwestern China.

Factor	Category	No. tested	No. positive	(%)(95% CI)	*p*-Value
Region	Kangding	201	51	25.37 (0.193–0.314)	
	Qionglai	15	4	26.67 (0.013–0.520)	<0.01
	Yaan	50	28	56.00 (0.417–0.703)	
Age (years)	0–1	183	61	33.33 (0.264–0.402)	0.318
	1–2	53	22	41.51 (0.278–0.552)	
Gender	Male	82	47	57.32 (0.464–0.683)	0.003
	Female	184	36	19.57 (0.138–0.254)	
Total		266	83	31.20 (0.256–0.368)	

### DNA extraction

Before conducting DNA extraction, potassium dichromate was removed from the fecal samples with distilled water by centrifugation for 10 min at 1500 ×*g*, three times. Genomic DNA was extracted from 200 mg of washed fecal matter using the EZNA1 Stool DNA kit (Omega Biotek, Norcross, GA, USA). Prior to use in PCR analysis, DNA was stored and frozen at −20 °C.

### PCR amplification

*Enterocytozoon bieneusi* genotypes were determined using a nested PCR amplification of the entire ITS region, and positive specimens were further detected by MLST analyses using the MS1, MS3, MS4, and MS7 loci. The primers and cycling parameters implemented for these reactions were as previously described [9, 37]. Negative controls were included in all PCR analyses. The secondary PCR products were subjected to electrophoresis in a 1.5% agarose gel and visualized under UV light by staining the gel with GoldView (Solarbio, China).

### Nucleotide sequencing and phylogenetic analysis

Secondary PCR amplicons of anticipated size were sequenced in both directions by Life Technologies (Guangzhou, China) with an ABI 3730DNA Analyzer (Applied Biosystems, Foster City, CA, USA) using the BigDye^®^ Terminator v3.1 cycle sequencing kit. Sequence accuracy was confirmed by bidirectional sequencing, and new PCR secondary products were re-sequenced, if necessary. To identify the *E. bieneusi* genotype, the sequences generated were respectively aligned with known reference sequences using BLAST and ClustalX 1.83. Mega 7.0 was used to construct the phylogenetic tree using the neighbor-joining (NJ) method (the Kimura two parameter model) with 1000 bootstrap replicates [17]. Novel genotype(s) of *E. bieneusi* were named according to the established system of nomenclature [34].

### Statistical analysis

The variations in *E. bieneusi* infection rates in Tibetan pigs between different areas, gender, and ages were compared using the Chi-square test. All tests were two-sided, with *p* < 0.05 indicating statistical significance. SPSS version 22.0 was used on all data. 95% confidence intervals (95% CIs) were calculated to explore the strength of the association between *E. bieneusi* occurrence and each factor.

### Nucleotide sequence accession numbers

Representative nucleotide sequences of *E. bieneusi* isolates were deposited in GenBank under accession numbers from MG581429 to MG581432 for ITS sequences and MH142189–MH142213 for the microsatellite (MS1, MS3, and MS7) and minisatellite (MS4) loci.

## Results and discussion

In the present study, of the 266 Tibetan pigs sampled, 83 were PCR-positive for *E. bieneusi*. Infection rates detected in Tibetan pigs were 25.4%, 56% and 26.6% in Kangding, Yaan and Qionglai, respectively. Differences between the three areas were significant (*χ* ^2^ = 17.648, *df* = 2, *p* < 0.01) (Table 1). In addition, the male Tibetan pig groups (17.7%, 47/266) had higher *E. bieneusi* prevalence than the female groups (13.5%, 36/266). The difference in the infection rate was also significant (*χ* ^2^ = 8.906, *df* = 1, *p* = 0.003). Although high infection rates were observed in 1–2 year-old pigs (41.51%, 22/53) and 0–1 year-olds (33.33%, 61/183), these rates were not significantly different (*χ* ^2^ = 1.240, *df* = 1, *p* > 0.05). The results of the present paper were previously published as a preprint [26]. With an overall infection rate of 31.2%, this rate is lower than the documented prevalence of *E. bieneusi* for wild boars in Sichuan province, China (41.2%), pigs in Henan province, China (45.5%), wild boars in central Europe (33.3%), and pigs in the State of Rio de Janeiro, Brazil (59.3%) [10, 24, 28, 46]. However, infection rates recorded in this study were higher than those for pigs in Guangdong province, China (26.39%), central Thailand (28.1%), and Japan (30%) [1, 30, 60]. Differences in infection rates between these studies may be largely attributable to climate and farming modes. Prevalence also varied across sample sites. Kangding, the only site on the Western Sichuan Plateau, had a prevalence of 25.4%, possibly reflecting the area’s high temperatures, and UV radiation, which may limit survival of *E. bieneusi s*pores and reduce transmission. Other factors influencing infection levels may include geo-ecological conditions, feeding/herd densities, herd management, sample size, and the condition of host animals. Differences in prevalence in Tibetan pigs between Yaan and Kangding are thought to reflect differences between traditional and modern herd management and breeding technologies.

Nucleotide sequences from ITS-PCR were obtained from the 83 *E. bieneusi*-positive specimens. The epidemiology and genotypes of *E. bieneusi* in different areas are given in Table 2. Four genotypes were detected, including two known genotypes (EbpC, Henan-IV) and two novel genotypes, which were named SCT01 and SCT02. Genotype EbpC was the most prevalent (21.8%, 58/266), and was detected in samples from all three cities. Genotype Henan-IV was only found in Kang ding (8.6%, 23/266). The novel genotypes SCT01 (0.3%, 1/266) and SCT02 (0.3%, 1/266) were only found in single specimens, both of which came from Yaan, and are the first newly-detected *E. bieneusi* genotypes from Tibetan pigs. Of the four genotypes identified in this study, EbpC was the most prevalent (69.9%, 58/83), and has been found in a number of animals, including cattle, dogs, cats, birds, non-human primates, bears, squirrels, sheep, foxes, deer, and humans [4, 8, 29, 36, 38, 39, 47, 51, 55]. EbpC is the prevalent *E. bieneusi* genotype associated with pig infection in China, reflecting *E. bieneusi*’s dominance as a porcine parasite. In addition, we also detected 26 records of Henan-IV (solely in Yaan), a zoonotic genotype associated with human infections in Henan province in China, and to date only recorded from China, where it demonstrates strict host specificity [44], occurring only in pigs and humans. To the best our knowledge, the two genotypes EbpC and Henan-IV were identified for the first time in Tibetan pigs in the present study. This species may be a key reservoir host of these genotypes (Table 4).

**Table 2.** Occurrence and genotypes of *E. bieneusi* in Tibetan pigs from different cities in southwest China.

Region	Farm ID	Prevalence (%)	Genotypes (*n*)
Kangding	Farm 1	31/102 (30.40)	EbpC (18), Henan-IV (*n* = 13)
	Farm 2	20/99 (20.20)	EbpC (12), Henan-IV (*n* = 8)
Yaan	Farm 3	14/28 (50.00)	EbpC (*n* = 14)
	Farm 4	10/22 (45.45)	EbpC (*n* = 12), SCT01 (*n* = 1), SCT02 (*n* = 1)
Qionglai	Farm 5	4/15 (26.67)	EbpC (*n* = 4)
Total		83/266 (31.20)	EbpC (58), Henan-IV (*n* = 23), SCT01 (*n* = 1), SCT02 (*n* = 1)

**Table 3.** Multilocus characterization of *Enterocytozoon bieneusi* isolates in Tibetan pigs in southwestern China.

ITS genotype	Multilocus genotype						No. of MLGs
	MS1	MS3	MS4	MS7	genbank accession nos.	MLGs	
Henan-IV	Type II*	Type I*	Type III	Type II	MH142190, MH142204, MH142200, MH142212	MLG1	1
Henan-IV	Type I	Type III*	Type III	Type II	MH142193, MH142205, MH142200, MH142212	MLG2	1
Henan-IV	Type I	Type I*	Type II*	Type I*	MH142193, MH142204, MH142199, MH142210	MLG3	1
Henan-IV	Type II*	Type II*	Type II*	Type II	MH142195, MH142206, MH142199, MH142212	MLG4	2
Ebpc	Type II*	Type I*	Type II*	Type II	MH142196, MH142204, MH142199, MH142209	MLG5	2
Ebpc	Type X*	Type I*	Type IV*	Type I*	MH142189, MH142204, MH142203, MH142213	MLG6	1
Ebpc	Type I	Type I*	Type II*	Type II	MH142193, MH142204, MH142199, MH142212	MLG7	1
Ebpc	Type II*	Type I*	Type I	Type IV*	MH142196, MH142204, MH142201, MH142213	MLG8	1
Ebpc	Type III*	Type IV*	Type I	Type III*	MH142194, MH142207, MH142201, MH142209	MLG9	1
Ebpc	Type I	Type IV*	Type I	Type II	MH142193, MH142207, MH142201, MH142209	MLG10	1

* Novel genotypes.

**Table 4.** Host ranges and geographical distribution of *Enterocytozoon bieneusi* genotypes in this study in China.

Genotype (synonym)	Host	Location	Isolate	Reference
EbpC (E, Peru4, WL13, WL17)	Pig	Shanghai	3	[9]
	Pig	Heilongjiang	10	[9]
	Pig	Heilongjiang	3	[21]
	Pig	Heilongjiang	3	[41]
	Pig	Jilin	1	[19]
	Pig	Mongolia	1	[19]
	Pig	Zhejiang	39	[45]
	Pig	Guangdong	17	[45]
	Pig	Yunnan	31	[45]
	Tibetan pig	Sichuan	58	This study
	Red panda	Shanxi	5	[40]
	Human	Shanghai	1	[42]
	Human	Henan	39	[43]
	Human	Heilongjiang	11	[48]
	Human, pig, monkey	Guangxi	4	[25]
	Squirrel	Sichuan	3	[4]
	Wild boar	Sichuan	85	[24]
	Nonhuman primates	Hebei	1	[16]
	Nonhuman primates	Hubei	3	[16]
	Nonhuman primates	Hunan	4	[16]
	Nonhuman primates	Being	2	[16]
	Nonhuman primates	Henan	5	[15]
	Water	Shanghai	37	[11]
	Wastewater	Shanghai	2	[18]
	Wastewater	Shanghai	2	[27]
	Wastewater	Shandong	1	[18]
	Wastewater	Hubei	5	[18]
	Camel	Xinjiang	23	[33]
	Fox	Heilongjiang	5	[61]
	Mink	Hebei	4	[53]
	Mink	Liaoning	3	[53]
	Mink	Jilin, Heilongjiang	6	[3]
	Chicken	Heilongjiang	2	[21]
	Flies	Henan	1	[49]
	Dog	Heilongjiang	2	[22]
	Dog	Shanxi	1	[14]
	Cattle	Henan, Ningxia	6	[23]
	Cattle	Hubei, Tianjin	1	[12]
	Goat	Yunnan	1	[2]
	Calve	Xinjiang	2	[32]
	Deer	Henan	4	[50]
	Deer	Henan	3	[13]
	Deer	Jilin	1	[13]
Henan-IV	Human	Henan	1	[43]
	Human	Heilongjiang	3	[48]
	Chicken	Jilin	2	[21]
	Camel	Xinjiang	1	[33]
	Horse	Xinjiang	21	[31]
	Cattle	Xinjiang	2	[32]
	Nonhuman primates	Hebei	2	[16]
	Nonhuman primates	Shanxi	1	[16]
	Nonhuman primates	Shanghai	1	[16]
	Pig	Heilongjiang	5	[41]
	Pig	Shanxi	3	[44]
	Pig	Yunnan	6	[45]
	Tibetan pig	Sichuan	23	This study
SCT01	Tibetan pig	Sichuan	1	This study
SCT02	Tibetan pig	Sichuan	1	This study

**Figure 1.**
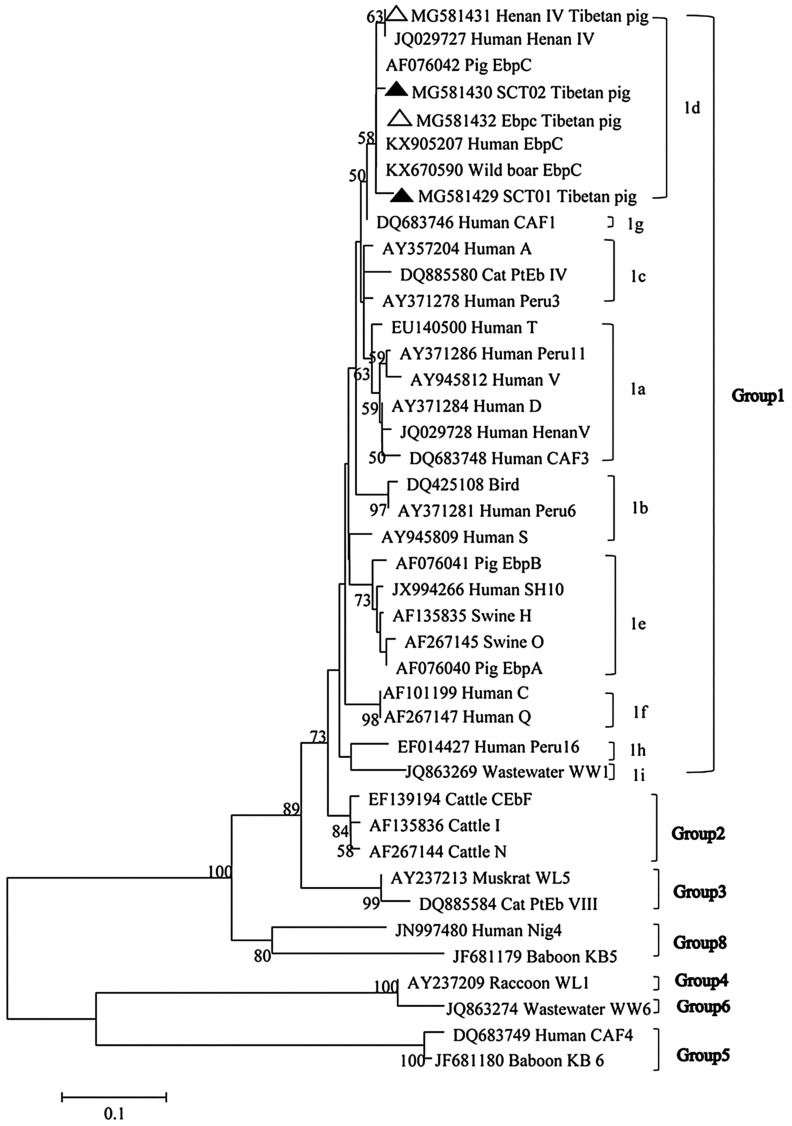
Phylogenetic relationship of *Enterocytozoon bieneusi* groups. The relationships between *E. bieneusi* genotypes identified in this study and other known genotypes deposited in the genbank were inferred by a neighbor-joining analysis of ITS sequences based on genetic distance by the Kimura-2-parameter model. The numbers on the branches represent percent bootstrapping values from 1000 replicates (only bootstrap values >50% are shown). Each sequence is identified by its accession number, genotype designation, and host origin. Genotypes with black triangles and open triangle are novel and known genotypes identified in this study, respectively.

Phylogenetic analysis based on ITS gene sequences of the four *E. bieneusi* genotypes obtained from the present study (two known and two novel genotypes) enabled classification for the genotypes as a single group (group 1), and further clustered into subgroup 1d, indicating zoonotic potential (Fig. 1). ITS gene sequence analysis revealed two novel genotypes, SCT01 (*n* = 1) and SCT02 (*n* = 1), both of which were detected in Yaan and clustered into group 1 zoonotic genotypes with public health significance. Other genotypes in this group include Henan-III in humans and EbpC from humans or wild boars [24, 43, 55]. Modes of transmission and zoonotic potential of *E. bieneusi* genotypes remain poorly known, and further molecular epidemiology studies are required. MLST holds promise for ongoing investigation of *E. bieneusi* taxonomy and genetic diversity [9]. Positive specimens were further characterized by PCR analyses of MS4, MS1, MS3 and MS7 to improve taxonomy and population genotypes of *E. bieneusi.* In all, 47, 48, 23 and 47 *E. bieneusi* isolates were amplified at the MS1, MS3, MS4, and MS7 loci, respectively, but only 12 samples were PCR-positive simultaneously at all four loci. Four distinct MLGs were observed in Henan-IV and six distinct MLGs in EbpC, named MLG1-4 and MLG5-10, respectively (Table 3). Nine, five, three and four novel types were detected at MS1, MS3, MS4 and MS7 loci, respectively. Analysis of 12 samples at four gene loci identified eight novel MLGs, including three genotype EbpC MLGs and five genotype Henan-IV MLGs (Table 3). These results reveal high genetic diversity in the Henan-IV and EbpC genotypes of *E. bieneusi* in Tibetan pigs.

## Conclusions

This study revealed an average *E. bieneusi* infection rate of 31.2% in three cities in Sichuan province, and is the first report of EbpC and Henan-IV in Tibetan pigs in China. Genetic diversity was characterized using MLST, and ten MLGs were identified. These results identify Tibetan pigs as possible vectors for zoonotic transmission of human microsporidiosis. Tibetan pigs are bred widely and there is frequent human contact, making them a significant public health risk in southwest China. Thus, measures are needed to control the transmission of *E. bieneusi* and to develop effective vaccines and drugs for use in the event of widespread human microsporidiosis.

## References

[1] Abe N, Kimata I. 2010 Molecular survey of *Enterocytozoon bieneusi* in a Japanese porcine population. Vector Borne & Zoonotic Diseases, 10, 425. 19725762 10.1089/vbz.2009.0039

[2] Chen D, Wang SS, Zou Y, Li Z, Xie SC, Shi LQ, Zou FC, Zhu XQ, Yang JF, Zhao GH. 2018 Prevalence and multi-locus genotypes of *Enterocytozoon bieneusi* in black-boned sheep and goats in Yunnan Province, southwestern China. Infection, Genetics and Evolution, 65, 385–391, S1567134818306397. 10.1016/j.meegid.2018.08.022 30145389

[3] Cong W, Qin SY, Meng QF. 2018 Molecular characterization and new genotypes of *Enterocytozoon bieneusi* in minks (Neovison vison) in China. Parasite, 25, 34. 30028674 10.1051/parasite/2018038 PMC6054522

[4] Deng L, Li W, Yu X, Gong C, Liu X, Zhong Z, Xie N, Lei S, Yu J, Fu H, Chen H, Xu H, Hu Y, Peng G. 2016 First report of the human-pathogenic *Enterocytozoon bieneusi* from Red-Bellied Tree Squirrels (*Callosciurus erythraeus*) in Sichuan, China. PLoS One, 11, e0163605. 27683278 10.1371/journal.pone.0163605 PMC5040432

[5] Deng L, Li W, Zhong Z, Gong C, Liu X, Huang X, Xiao L, Zhao R, Wang W, Feng F. 2016 Molecular characterization and multilocus genotypes of *Enterocytozoon bieneusi* among horses in southwestern China. Parasites & Vectors, 9, 561. 27776551 10.1186/s13071-016-1844-3 PMC5078948

[6] Didier ES, Weiss LM. 1969 Microsporidiosis: not just in AIDS patients. Current Opinion in Infectious Diseases, 24, 490–495. 10.1097/QCO.0b013e32834aa152 PMC3416021 21844802

[7] Didier ES, Weiss LM. 2006 Microsporidiosis: current status. Current Opinion in Infectious Diseases, 19, 485. 16940873 10.1097/01.qco.0000244055.46382.23 PMC3109650

[8] Ding S, Huang W, Qin Q, Tang J, Liu H. 2018 Genotype identification and phylogenetic analysis of *Enterocytozoon bieneusi* isolates from stool samples of diarrheic children. Journal of Parasitology, 104, 297. 29457962 10.1645/17-108

[9] Feng Y, Li N, Dearen T, Lobo ML, Matos O, Cama V, Xiao L. 2011 Development of a multilocus sequence typing tool for high-resolution genotyping of *Enterocytozoon bieneusi*. Applied & Environmental Microbiology, 77, 4822–4828. 21622791 10.1128/AEM.02803-10 PMC3147401

[10] Fiuza VR, Oliveira FC, Fayer R, Santín M. 2015 First report of *Enterocytozoon bieneusi* in pigs in Brazil. Parasitology International, 64, 18–23. 10.1016/j.parint.2015.01.002 25582928

[11] Hu Y, Feng Y, Huang C, Xiao L. 2014 Occurrence, source, and human infection potential of *Cryptosporidium* and *Enterocytozoon bieneusi* in drinking source water in Shanghai, China, during a pig carcass disposal incident. Environmental Science & Technology, 48, 14219–14227. 25383482 10.1021/es504464t PMC5788171

[12] Hu S, Liu Z, Yan F, Zhang Z, Zhang G, Zhang L, Jian F, Zhang S, Ning C, Wang R. 2017 Zoonotic and host-adapted genotypes of *Cryptosporidium* spp., *Giardia duodenalis* and *Enterocytozoon bieneusi* in dairy cattle in Hebei and Tianjin, China. Veterinary Parasitology, 248, 68–73. 29173544 10.1016/j.vetpar.2017.10.024

[13] Huang J, Zhang Z, Yang Y, Wang R, Zhao J, Jian F, Ning C, Zhang L. 2017 New genotypes of *Enterocytozoon bieneusi* isolated from Sika Deer and Red Deer in China. Frontiers in Microbiology, 8, 879. 28572794 10.3389/fmicb.2017.00879 PMC5435827

[14] Karim MR, Dong H, Li T, Yu F, Li D, Zhang L, Li J, Wang R, Li S, Li X. 2015 Predomination and new genotypes of *Enterocytozoon bieneusi* in captive nonhuman primates in zoos in China: high genetic diversity and zoonotic significance. Plos One, 10, e0117991. 25705879 10.1371/journal.pone.0117991 PMC4338232

[15] Karim MR, Dong H, Yu F, Jian F, Zhang L, Wang R, Zhang S, Rume FI, Ning C, Xiao L. 2014 Genetic diversity in *Enterocytozoon bieneusi* isolates from dogs and cats in China: host specificity and public health implications. Journal of Clinical Microbiology, 52, 3297–3302. 24989604 10.1128/JCM.01352-14 PMC4313153

[16] Karim MR, Wang R, Dong H, Zhang L, Li J, Zhang S, Rume FI, Qi M, Jian F, Sun M, Yang G, Zou F, Ning C, Xiao L. 2014 Genetic polymorphism and zoonotic potential of *Enterocytozoon bieneusi* from nonhuman primates in China. Applied & Environmental Microbiology, 80, 1893. 24413605 10.1128/AEM.03845-13 PMC3957649

[17] Kumar S, Stecher G, Tamura K. 2016 MEGA7: molecular evolutionary genetics analysis version 7.0 for bigger datasets. Molecular Biology and Evolution, 33, 1870–1874. 27004904 10.1093/molbev/msw054 PMC8210823

[18] Li N, Xiao L, Wang L, Zhao S, Zhao X, Duan L, Guo M, Liu L, Feng Y. 2012 Molecular surveillance of *Cryptosporidium spp.*, *Giardia duodenalis*, and *Enterocytozoon bieneusi* by genotyping and subtyping parasites in wastewater. PLoS Neglected Tropical Diseases, 6, e1809. 22970334 10.1371/journal.pntd.0001809 PMC3435239

[19] Li W, Diao R, Yang Xiao L, Lu Y, Li Y, Song M. 2014 High diversity of human-pathogenic *Enterocytozoon bieneusi* genotypes in swine in northeast China. Parasitology Research, 113, 1147. 24442159 10.1007/s00436-014-3752-9

[20] Li W, Li Y, Li W, Yang J, Song M, Diao R, Jia H, Lu Y, Zheng J, Zhang X. 2014 Genotypes of *Enterocytozoon bieneusi* in livestock in China: high prevalence and zoonotic potential. Plos One, 9, e97623. 24845247 10.1371/journal.pone.0097623 PMC4028308

[21] Li W, Tao W, Jiang Y, Diao R, Yang J, Xiao L. 2014 Genotypic distribution and phylogenetic characterization of *Enterocytozoon bieneusi* in diarrheic chickens and pigs in multiple cities, China: potential zoonotic transmission. Plos One, 9, e108279. 25255117 10.1371/journal.pone.0108279 PMC4177920

[22] Li W, Li Y, Song M, Lu Y, Yang J, Tao W, Jiang Y, Wan Q, Zhang S, Xiao L. 2015 Prevalence and genetic characteristics of *Cryptosporidium*, *Enterocytozoon bieneusi* and *Giardia duodenalis* in cats and dogs in Heilongjiang province, China. Veterinary Parasitology, 208, 125–134. 25665462 10.1016/j.vetpar.2015.01.014

[23] Li J, Luo N, Wang C, Qi M, Cao J, Cui Z, Huang J, Wang R, Zhang L. 2016 Occurrence, molecular characterization and predominant genotypes of *Enterocytozoon bieneusi* in dairy cattle in Henan and Ningxia, China. Parasites & Vectors, 9, 1–5. 26968376 10.1186/s13071-016-1425-5 PMC4788901

[24] Li W, Deng L, Wu K, Huang X, Song Y, Su H, Hu Y, Fu H, Zhong Z, Peng G. 2017 Presence of zoonotic *Cryptosporidium scrofarum*., *Giardia duodenalis* assemblage A and *Enterocytozoon bieneusi* genotypes in captive Eurasian wild boars (*Sus scrofa*) in China: potential for zoonotic transmission. Parasites & Vectors, 10, 10. 28061911 10.1186/s13071-016-1942-2 PMC5219718

[25] Liu H, Jiang Z, Yuan Z, Yin J, Wang Z, Yu B, Zhou D, Shen Y, Cao J. 2017 Infection by and genotype characteristics of *Enterocytozoon bieneusi* in HIV/AIDS patients from Guangxi Zhuang autonomous region, China. BMC Infectious Diseases, 17, 684. 29029610 10.1186/s12879-017-2787-9 PMC5640944

[26] Luo R, Xiang L, Liu H, Zhong Z, Liu L, Deng L, Song Y, Liu L, Huang X, Zhou Z, Fu H, Luo Y. 2018 First report and multilocus genotyping of *Enterocytozoon bieneusi* from Tibetan pigs in southwestern China. 10.1101/327767. PMC6492536 31041895

[27] Ma J, Feng Y, Hu Y, Villegas EN, Xiao L. 2016 Human infective potential of *Cryptosporidium* spp., *Giardia duodenalis* and *Enterocytozoon bieneusi* in urban wastewater treatment plant effluents. Journal of Water & Health, 14, 411–423. 27280607 10.2166/wh.2016.192 PMC5788172

[28] Nemejc K, Sak B, Kvetonová D, Hanzal V, Janiszewski P, Forejtek P, Rajský D, Kotková M, Ravaszová P, Mcevoy J. 2014 Prevalence and diversity of *Encephalitozoon* spp. and *Enterocytozoon bieneusi* in wild boars (*Sus scrofa*) in Central Europe. Parasitology Research, 113, 761. 24292543 10.1007/s00436-013-3707-6

[29] Piekarska J, Kicia M, Wesołowska M, Kopacz Ż, Gorczykowski M, Szczepankiewicz B, Kvac M, Sak B. 2017 Zoonotic microsporidia in dogs and cats in Poland. Veterinary Parasitology, 246, 108–111. 28969771 10.1016/j.vetpar.2017.09.011

[30] Prasertbun R, Mori H, Pintong AR, Sanyanusin S, Popruk S, Komalamisra C, Changbunjong T, Buddhirongawatr R, Sukthana Y, Mahittikorn A. 2016 Zoonotic potential of *Enterocytozoon* genotypes in humans and pigs in Thailand. Veterinary Parasitology, 233, 73–79. 28043391 10.1016/j.vetpar.2016.12.002

[31] Qi M, Jing B, Jian F, Wang R, Zhang S, Wang H, Ning C, Zhang L. 2017 Dominance of *Enterocytozoon bieneusi* genotype J in dairy calves in Xinjiang, Northwest China. Parasitology International, 66, 960–963. 27794506 10.1016/j.parint.2016.10.019

[32] Qi M, Li J, Zhao A, Cui Z, Wei Z, Jing B, Zhang L. 2018 Host specificity of *Enterocytozoon bieneusi* genotypes in Bactrian camels (*Camelus bactrianus*) in China. Parasites & Vectors, 11, 219. 29609645 10.1186/s13071-018-2793-9 PMC5880058

[33] Qi M, Wang R, Wang H, Jian F, Li J, Zhao J, Dong H, Zhu H, Ning C, Zhang L. 2016 *Enterocytozoon bieneusi* genotypes in grazing horses in China and their zoonotic transmission potential. Journal of Eukaryotic Microbiology, 63, 591–597. 26909747 10.1111/jeu.12308

[34] Santin M, Fayer R. 2010 *Enterocytozoon bieneusi* genotype nomenclature based on the internal transcribed spacer sequence: a consensus. Journal of Eukaryotic Microbiology, 56, 34–38. 10.1111/j.1550-7408.2008.00380.x 19335772

[35] Santin M, Fayer R. 2011 Microsporidiosis: *Enterocytozoon bieneusi* in domesticated and wild animals. Research in Veterinary Science, 90, 363–371. 20699192 10.1016/j.rvsc.2010.07.014

[36] Shi K, Li M, Wang X, Li J, Karim MR, Wang R, Zhang L, Jian F, Ning C. 2016 Molecular survey of *Enterocytozoon bieneusi* in sheep and goats in China. Parasites & Vectors, 9, 23. 26782742 10.1186/s13071-016-1304-0 PMC5024852

[37] Sulaiman IM, Ronald F, Lal AA, Trout JM, Schaefer FW, Lihua X. 2003 Molecular characterization of microsporidia indicates that wild mammals harbor host-adapted *Enterocytozoon spp.* as well as human-pathogenic *Enterocytozoon bieneusi*. Applied & Environmental Microbiology, 69, 4495. 12902234 10.1128/AEM.69.8.4495-4501.2003 PMC169096

[38] Tang C, Cai M, Wang L, Guo Y, Li N, Feng Y, Xiao L. 2018 Genetic diversity within dominant *Enterocytozoon bieneusi* genotypes in pre-weaned calves. Parasites & Vectors, 11, 170. 29530084 10.1186/s13071-018-2768-x PMC5848593

[39] Tavalla M, Mardani-Kateki M, Abdizadeh R, Soltani S, Saki J. 2017 Molecular diagnosis of potentially human pathogenic *Enterocytozoon bieneusi* and *Encephalitozoon* species in exotic birds in Southwestern Iran. Journal of Infection and Public Health, 11, 192–196. 28869156 10.1016/j.jiph.2017.07.028

[40] Tian GR, Zhao GH, Du SZ, Hu XF, Wang HB, Zhang LX, Yu SK. 2015 First report of *Enterocytozoon bieneusi* from giant pandas (*Ailuropoda melanoleuca*) and red pandas (*Ailurus fulgens*) in China. Infection, Genetics and Evolution, 34, 32–35. 10.1016/j.meegid.2015.06.015 26079276

[41] Wan Q, Lin Y, Mao Y, Yang Y, Li Q, Zhang S, Jiang Y, Tao W, Li W. 2016 High prevalence and widespread distribution of zoonotic *Enterocytozoon bieneusi* genotypes in swine in Northeast China: implications for public health. Journal of Eukaryotic Microbiology, 63, 162–170. 26333563 10.1111/jeu.12264

[42] Wang L, Xiao L, Duan L, Ye J, Guo Y, Guo M, Liu L, Feng Y. 2013 Concurrent infections of *Giardia duodenalis*, *Enterocytozoon bieneusi*, and *Clostridium difficile* in children during a cryptosporidiosis outbreak in a pediatric hospital in China. PLoS Neglected Tropical Diseases, 7(9), e2437. 24069491 10.1371/journal.pntd.0002437 PMC3772047

[43] Wang L, Zhang H, Zhao X, Zhang L, Zhang G, Guo M, Liu L, Feng Y, Xiao L. 2013 Zoonotic *Cryptosporidium* species and *Enterocytozoon bieneusi* genotypes in HIV-positive patients on antiretroviral therapy. Journal of Clinical Microbiology, 51, 557–563. 23224097 10.1128/JCM.02758-12 PMC3553929

[44] Wang SS, Li JQ, Li YH, Wang XW, Fan XC, Liu X, Li ZJ, Song JK, Zhang LX, Zhao GH. 2018 Novel genotypes and multilocus genotypes of *Enterocytozoon bieneusi* in pigs in northwestern China: a public health concern. Infection, Genetics and Evolution, 63, 89–94. 10.1016/j.meegid.2018.05.015 29792989

[45] Wang SS, Wang RJ, Fan XC, Liu TL, Zhang LX, Zhao GH. 2018 Prevalence and genotypes of *Enterocytozoon bieneusi* in China. Acta Tropica, 183, 142–152. 29660311 10.1016/j.actatropica.2018.04.017

[46] Wang H, Zhang Y, Wu Y, Li J, Qi M, Li T, Wang J, Wang R, Zhang S, Jian F. 2018 Occurrence, molecular characterization and assessment of zoonotic risk of *Cryptosporidium* spp., *Giardia duodenalis*, and *Enterocytozoon bieneusi* in pigs in Henan, Central China. Journal of Eukaryotic Microbiology, 65, 893–901. 29752883 10.1111/jeu.12634

[47] Wu J, Han JQ, Shi LQ, Zou Y, Li Z, Yang JF, Huang CQ, Zou FC. 2018 Prevalence, genotypes, and risk factors of *Enterocytozoon bieneusi* in Asiatic black bear (*Ursus thibetanus*) in Yunnan Province, Southwestern China. Parasitology Research, 117, 1–7. 29450634 10.1007/s00436-018-5791-0

[48] Yang J, Song M, Wan Q, Li Y, Lu Y, Jiang Y, Tao W, Li W. 2014 *Enterocytozoon bieneusi* genotypes in children in Northeast China and assessment of risk of zoonotic transmission. Journal of Clinical Microbiology, 52, 4363. 25274994 10.1128/JCM.02295-14 PMC4313317

[49] Yu F, Qi M, Zhao Z, Lv C, Wang Y, Wang R, Zhang L. 2018 The potential role of synanthropic rodents and flies in the transmission of *Enterocytozoon bieneusi* on a dairy cattle farm in China. Journal of Eukaryotic Microbiology. 10.1111/jeu.12687. 30191674

[50] Zhang Z, Huang J, Karim MR, Zhao J, Dong H, Ai W, Li F, Zhang L, Wang R. 2015 Zoonotic *Enterocytozoon bieneusi* genotypes in Pere David’s deer (*Elaphurus davidianus*) in Henan, China. Experimental Parasitology, 155, 46–48. 25982030 10.1016/j.exppara.2015.05.008

[51] Zhang XX, Cong W, Lou ZL, Ma JG, Zheng WB, Yao QX, Zhao Q, Zhu XQ. 2016 Prevalence, risk factors and multilocus genotyping of *Enterocytozoon bieneusi* in farmed foxes (*Vulpes lagopus*), Northern China. Parasites & Vectors, 9, 1–7. 26847241 10.1186/s13071-016-1356-1 PMC4743323

[52] Zhang Q, Cai J, Li P, Wang L, Guo Y, Li C, Lei M, Feng Y, Xiao L. 2018 *Enterocytozoon bieneusi* genotypes in Tibetan sheep and yaks. Parasitology Research, 117, 1–7. 29332156 10.1007/s00436-017-5742-1

[53] Zhang XX, Jiang RL, Ma JG, Xu C, Zhao Q, Hou G, Liu GH. 2018 *Enterocytozoon bieneusi* in Minks (*Neovison vison*) in Northern China: a Public Health Concern. Frontiers in Microbiology, 9, 1221. 29946304 10.3389/fmicb.2018.01221 PMC6005834

[54] Zhang Y, Koehler AV, Wang T, Haydon SR, Gasser RB. 2018 First detection and genetic characterisation of *Enterocytozoon bieneusi* in wild deer in Melbourne’s water catchments in Australia. Parasites & Vectors, 11, 2. 29295716 10.1186/s13071-017-2577-7 PMC5751821

[55] Zhong Z, Li W, Deng L, Song Y, Wu K, Tian Y, Huang X, Hu Y, Fu H, Geng Y. 2017 Multilocus genotyping of *Enterocytozoon bieneusi* derived from nonhuman primates in southwest China. Plos One, 12, e0176926. 28498867 10.1371/journal.pone.0176926 PMC5428909

[56] Zhong Z, Tian Y, Song Y, Deng L, Li J, Ren Z, Ma X, Gu X, He C, Geng Y. 2017 Molecular characterization and multi-locus genotypes of *Enterocytozoon bieneusi* from captive red kangaroos (*Macropus Rfus*) in Jiangsu province, China. Plos One, 12, e0183249. 28806735 10.1371/journal.pone.0183249 PMC5555684

[57] Zhao W, Zhang W, Yang F, Cao J, Liu H, Yang D, Shen Y, Liu A. 2014 High prevalence of *Enterocytozoon bieneusi* in asymptomatic pigs and assessment of zoonotic risk at the genotype level. Applied & Environmental Microbiology, 80, 3699–3707. 24727270 10.1128/AEM.00807-14 PMC4054152

[58] Zhao GH, Du SZ, Wang HB, Hu XF, Deng MJ, Yu SK, Zhang LX, Zhu XQ. 2015 First report of zoonotic *Cryptosporidium* spp., *Giardia intestinalis* and *Enterocytozoon bieneusi* in golden takins (*Budorcas taxicolor bedfordi*). Infection, Genetics and Evolution, 34, 394–401. 10.1016/j.meegid.2015.07.016 26190449

[59] Zhao W, Wang J, Yang Z, Liu A. 2017 Dominance of the *Enterocytozoon bieneusi* genotype BEB6 in red deer (*Cervus elaphus*) and Siberian roe deer (*Capreolus pygargus*) in China and a brief literature review. Parasite, 24, 54. 29267159 10.1051/parasite/2017056 PMC5739546

[60] Zou Y, Hou JL, Li FC, Zou FC, Lin RQ, Ma JG, Zhang XX, Zhu XQ. 2018 Prevalence and genotypes of *Enterocytozoon bieneusi* in pigs in southern China. Infection, Genetics and Evolution, 66, 52–56. 10.1016/j.meegid.2018.09.006 30218706

[61] Zhao W, Zhang W, Yang Z, Liu A, Zhang L, Yang F, Wang R, Ling H. 2015 Correction: Genotyping of *Enterocytozoon bieneusi* in farmed Blue Foxes (*Alopex lagopus*) and Raccoon Dogs (*Nyctereutes procyonoides*) in China. Plos One, 10(11), e0143992. 26544711 10.1371/journal.pone.0142611 PMC4636423

